# Componential Analysis of English Verbs

**DOI:** 10.3389/frai.2022.780385

**Published:** 2022-05-30

**Authors:** Ghazaleh Kazeminejad, Martha Palmer, Susan Windisch Brown, James Pustejovsky

**Affiliations:** ^1^Department of Linguistics, University of Colorado Boulder, Boulder, CO, United States; ^2^Department of Computer Science, Brandeis University, Waltham, MA, United States

**Keywords:** componential analysis, lexical semantics, verb lexicon, logical predicates, VerbNet

## Abstract

Computational lexical resources such as WordNet, PropBank, VerbNet, and FrameNet are in regular use in various NLP applications, assisting in the never-ending quest for richer, more precise semantic representations. Coherent class-based organization of lexical units in VerbNet and FrameNet can improve the efficiency of processing by clustering similar items together and sharing descriptions. However, class members are sometimes quite different, and the clustering in both can gloss over useful fine-grained semantic distinctions. FrameNet officially eschews syntactic considerations and focuses primarily on semantic coherence, associating nouns, verbs and adjectives with the same semantic frame, while VerbNet considers both syntactic and semantic factors in defining a class of verbs, relying heavily on meaning-preserving diathesis alternations. Many VerbNet classes significantly overlap in membership with similar FrameNet Frames, e.g., VerbNet *Cooking-45.3* and FrameNet Apply_heat, but some VerbNet classes are so heterogeneous as to be difficult to characterize semantically, e.g., *Other_cos-45.4*. We discuss a recent addition to the VerbNet class semantics, verb-specific semantic features, that provides significant enrichment to the information associated with verbs in each VerbNet class. They also implicitly group together verbs sharing semantic features within a class, forming more semantically coherent subclasses. These efforts began with introspection and dictionary lookup, and progressed to automatic techniques, such as using NLTK sentiment analysis on verb members of VerbNet classes with an Experiencer argument role, to assign positive, negative or neutral labels to them. More recently we found the Brandeis Semantic Ontology (BSO) to be an invaluable source of rich semantic information and were able to use a VerbNet-BSO mapping to find fine-grained distinctions in the semantic features of verb members of 25 VerbNet classes. This not only confirmed the assignments previously made to classes such as *Admire-31.2*, but also gave a more fine-grained semantic decomposition for the members. Also, for the *Judgment-31.1* class, the new method revealed new, more fine-grained existing semantic features for the verbs. Overall, the BSO mapping produced promising results, and as a manually curated resource, we have confidence the results are reliable and need little (if any) further hand-correction. We discuss our various techniques, illustrating the results with specific classes.

## 1. Introduction

What do we mean by lexical semantics? Just like any other question, the answer to this depends on the theory and the standpoint from which we look at the question or problem. The standpoint we adopt in this paper focuses on layers of meaning that distinguish between two similar words. For instance, what distinguishes the words “man” and “boy” is the feature of age—whether the male referent is an adult (therefore a man) or not (therefore a boy). This view of lexical semantics targets the semantic components of words, trying to find how the meanings of two words are perceived as different based on their componential analysis. This view has a long history which can be traced back at least to Aristotle and Socrates, and the deep-seated ontological method of dividing a genus into species and species into sub-species (Lipka, 1979). It is this analytical method that is employed by dictionaries in presenting the meaning of words. For example, Merriam-Webster Online Dictionary defines the motion sense of the verb “run” as “to go faster than a walk”. So “run” indicates a motion event where speed is a distinguishing feature. Hence, the highlighted semantic component in this verb-pair distinction (“run” vs. “walk”) is about *speed*.

Just like phonemes that can create two separate words that we know as a minimal pair (e.g., /d/ and /ð/ in words “day” and “they”, a difference that is not recognized in many other languages), semantic components, or semantic features, might be different from one language to another. For instance, one of the semantic features involved in composing verbs in English is the *manner* component. As an example, the verb “glide” in the Merriam-Webster Online Dictionary is defined as: to move smoothly, continuously, and effortlessly. Apparently, this verb indicates motion in a particular manner. Another example, this time from the nominal domain, is in naming animals, where the semantic primitives of age and gender play a role, but not size or color (e.g., Rooster vs. Hen vs. Chick, and Drake vs. Duck vs. Duckling). (Gentner, 1981) shows that the notion of componential representation of lexical meaning can be useful in explaining a number of psycholinguistic phenomena, and concludes that accessing componential lexical semantic representations is an important aspect of comprehension. fMRI-based neurolinguistic studies (Kemmerer et al., 2008) have also confirmed the existence of semantic components in English verbs, proving that the five semantic components they focused on (including action, motion, contact, change of state, and tool use) are processed in different regions of the brain. A recent effort in psycholinguistics has successfully explored the use of human judgements to confirm the validity of the underlying semantic concepts such as exertion of force or change of state that are associated with VerbNet classes (Hartshorne et al., 2013, 2014).

This componential view of lexical semantics, particularly as applied to the domain of verbs (Hartshorne et al., 2013, 2014) has been utilized in the semantic representation of English verbs in the VerbNet lexical resource (Schuler, 2005; Brown et al., 2018, 2019, 2022), in the sense that English verbs have been assigned to different classes based on their semantic (and syntactic) differences, which entails that all the verbs assigned to the same class share some aspects of their semantics. Under development since 1998 (Dang et al., 1998; Schuler, 2005), VerbNet has richer and more detailed representations than PropBank (Kingsbury and Palmer, 2002; Palmer et al., 2005) and its coverage of verbs is more extensive than that of FrameNet (Baker et al., 1998). It also contains mappings to those resources as well as OntoNotes (Hovy et al., 2006), WordNet (Miller, 1995), and Event Force Dynamics (Kalm et al., 2019). It is worth noting that one of the factors that distinguishes VerbNet from other similar resources is the manually curated semantic representations for verb classes in the form of logical predicates^1^, which are shared among all the verb members of the class. In its most recent version (Brown et al., 2018, 2019, 2022), VerbNet has adopted the dynamic event structure ideas introduced by the Generative Lexicon theory (Pustejovsky, 1998, 2013; Pustejovsky and Moszkowicz, 2011). These recent changes have also been aimed at facilitating the use of VerbNet by AI planners (McDonald et al., 2018).

VerbNet is based on Beth Levin's (1993) lexical semantic theory, which postulated that differences in verbs' affinities for syntactic alternations reflect differences in their semantics. Some of the classes use alternations that reflect very broad semantic qualities. For example, many verbs that in some way indicate a change of state, *cos*, are stacked in one class (*Other_cos-45.4*) based on the fact that they can participate in similar syntactic frames. However, semantically, these verbs can be further divided into a number of subclasses based on what type of change they indicate, including (but not limited to) verbs that indicate creation or complete annihilation of an object, verbs that indicate abstract changes, or physical or chemical changes, changes in temperature, size, color, etc., and verbs that indicate a certain direction of change, such as increase, decrease, or fluctuation.

If Levin's theory and distributional language models have one thing in common, it is the fact that both rely on syntax to a certain extent to capture semantics. One of Levin's main criteria for class membership assignment for a verb is based on the specific syntactic alternations in which the verb can participate. This has distracted from the differences in semantics of the verbs in a given class. Similarly, distributional semantics tries to capture the semantics of words based on their patterns of occurrence in a text corpus, which often takes into consideration syntactic information such as position and function words as well as neighboring content words. Linguistically, syntactic structural information is especially relevant for an analytic language such as English, where syntax plays an especially important role. However, there are some aspects of semantics that systems relying on surface structure fail to capture. For example, when language models encounter a change of state event, their prediction of the direction of change (increase, decrease, or fluctuation) is often inaccurate and unreliable. This is not surprising, since in all other respects the contexts of words like increase and decrease can be very similar. Depending on the intended application of the language model, inaccurate prediction of the positive/negative, increase/decrease direction of change could be detrimental. In addition, natural language inference (NLI) tasks need vast amounts of annotated text to achieve their goal. As we will see in Section 5, using the recently introduced verb-specific features can be beneficial to an NLI task.

Users of VerbNet drew our attention to the heterogeneous nature of many of the classes. For example, Gao et al. (2016) explore the causality of action verbs and their implied changes in the physical world. As an example, “slicing a pizza” results in the state of the PATIENT (pizza) changing from one big piece to several smaller pieces. They state that despite existing linguistic studies of physical causality, lexicons do not present a detailed account of physical causality denoted by an action verb (see Table 1 for categories of physical causality introduced by Gao et al., 2016). In particular, they mention VerbNet as a lexical resource that contains semantic representations for English verbs indicating some type of change of state involved for action verbs, but they point out that even VerbNet lacks details about what type of change is occurring with each verb. Their intended usage for physical causality was in modeling and state detection in grounded language understanding. Another work pointing out this deficit in VerbNet was Clark et al. (2018), which was an attempt to track the states of entities in a paragraph describing a process. They used VerbNet to build a rulebase of the preconditions and effects of actions. They successfully use VerbNet to capture changes in location and existence, but they suggest that they cannot go any further in capturing other types of change of state with VerbNet since it does not explicitly model size, temperature, or phase changes.

**Table 1:** Categories of physical causality (Gao et al., 2016).

**Type**	**Attribute**	**Attribute value**
Dimension	Size, length, volume	Changes, increases, decreases, specific
Color/Texture	Color	Appear, disappear, changes, mix, separate, specific (green, red, etc.)
Physical Property	Weight	Increase, decrease
Quantification	Number of pieces	Increases, one becomes many, decreases, many become one
Position	Location	Changes, enter/exit container, specific

We took on the challenge of subdividing these heterogeneous classes—an attempt that gave rise to a new layer of information for VerbNet which is still under development: verb-specific semantic features for the verbs in a class. By having shared membership of a class, it is already asserted for these verbs that they are similar to each other in terms of their semantics (i.e., what types of participants they assume, how they can be represented using logical predicates, etc.) and syntactic behavior (i.e., what types of syntactic alternations they can undergo, what types of syntactic frames they can occur in, including what types of valency they can have, etc.). For each verb in a given class, distinctive semantic features (i.e., those semantic features that distinguish between different verb members of a class) were extracted using different methods and represented as a new layer of information in the XML files. We found the Brandeis Semantic Ontology (Pustejovsky et al., 2006) to be a rich source of verb specific semantic features (see Sections 2.4, 3.3).

In this paper, we first describe the relevant resources used (see Section 2), and then we review the methods we used in this process (Section 3) and present the results (Section 4). We also describe how we have implemented them in the VerbNet XML files and the Unified Verb Index, a searchable website developed to display NLP resources including VerbNet, PropBank, OntoNotes, and FrameNet (Section 4.3.1). Section 5 discusses the results, and Section 6 concludes.

## 2. Resources

In this section, we begin with a detailed description of our primary resource, VerbNet, and then discuss additional resources that proved beneficial in adding verb-specific features.

### 2.1. VerbNet

**Figure 1:**
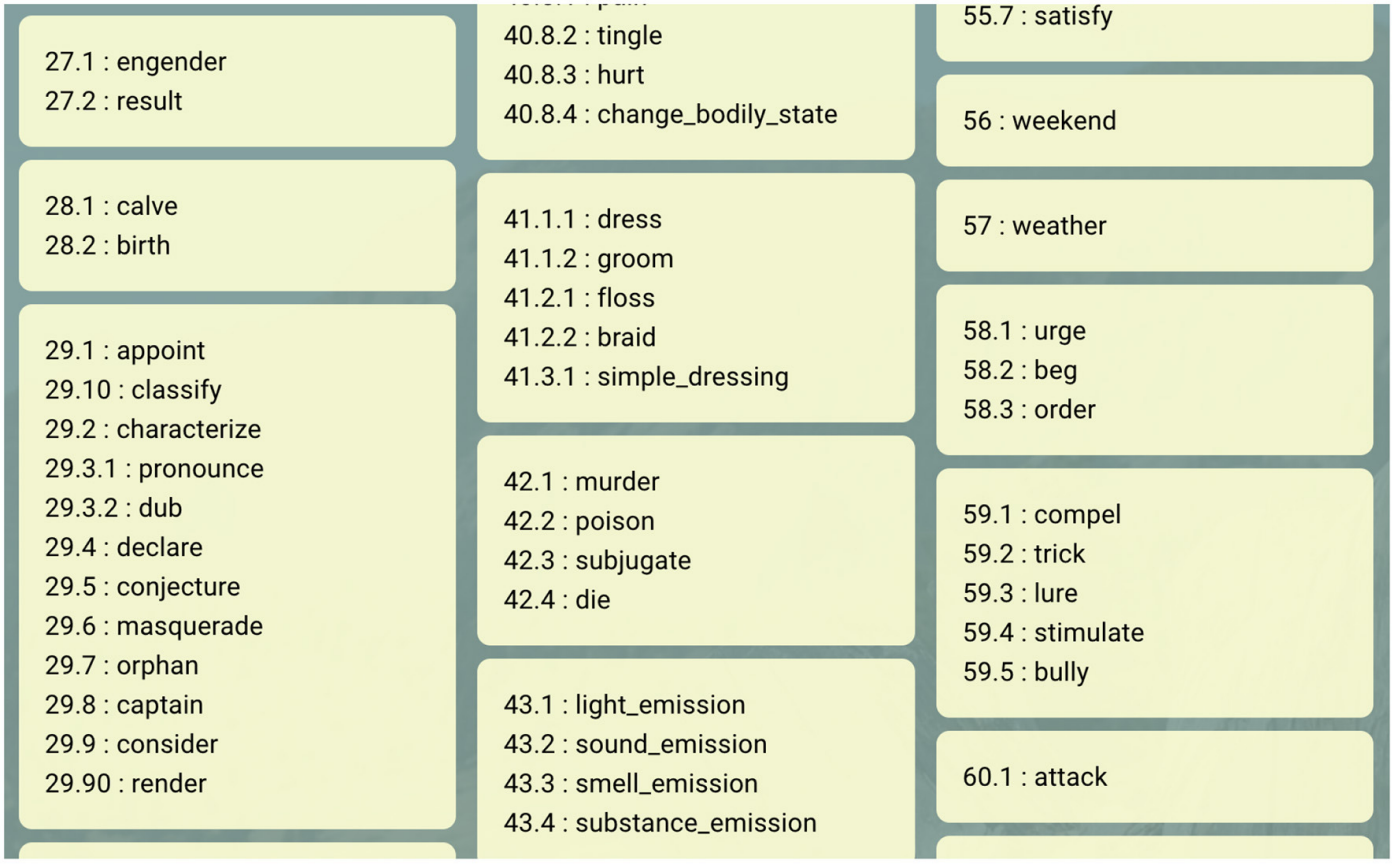
A screenshot of a subset of the VN class hierarchy, from the UVI.

VerbNet (Kipper et al., 2006) is a hierarchical, wide-coverage verb lexicon that was originally based on Levin's (1993) analysis of English verbs and has been expanded into dozens of additional classes and hundreds of additional verbs and verb senses. VerbNet cohesively clusters verbs into classes based on their similarities in syntactic and semantic behavior (Schuler, 2005). Each VerbNet class contains a set of member verbs, the thematic roles for the predicate-argument structure of these verbs, the selectional preferences for these class-specific roles, as well as a set of typical syntactic patterns and corresponding semantic representations. These semantic representations use a Davidsonian first-order-logic formulation to provide an abstract, language-independent conceptual representation of actions, such as changes of state, changes of location, and exertion of force (Brown et al., 2019, 2022). These representations use basic predicates to show the relationships between the thematic role arguments and to track any changes over the time course of the event. In terms of naming, the class nominal and numerical parts follow Levin's (1993) nomenclature. A list of VerbNet class names can be found at https://uvi.colorado.edu/class_hierarchy. The way the classes have been visualized on this page shows how the first number following the dash semantically clusters the classes sharing that number (see Figure 1). For instance, *Cut-21.1* and *Carve-21.2* share an AGENT who makes a PATIENT lose its material integrity by making an INSTRUMENT contact the PATIENT. The main semantic distinction between the two classes is that the verbs in the *Carve-22.2* class end up having a verb specific form—a fact that is represented in the **has_physical_form**(e3, Patient, V_Form) logical form. On the other hand, the *Cut-22.1* class contains a verb-specific movement manner by the AGENT, represented in the **manner**(e2, V_Movement, Agent) logical form.

**Figure 2:**
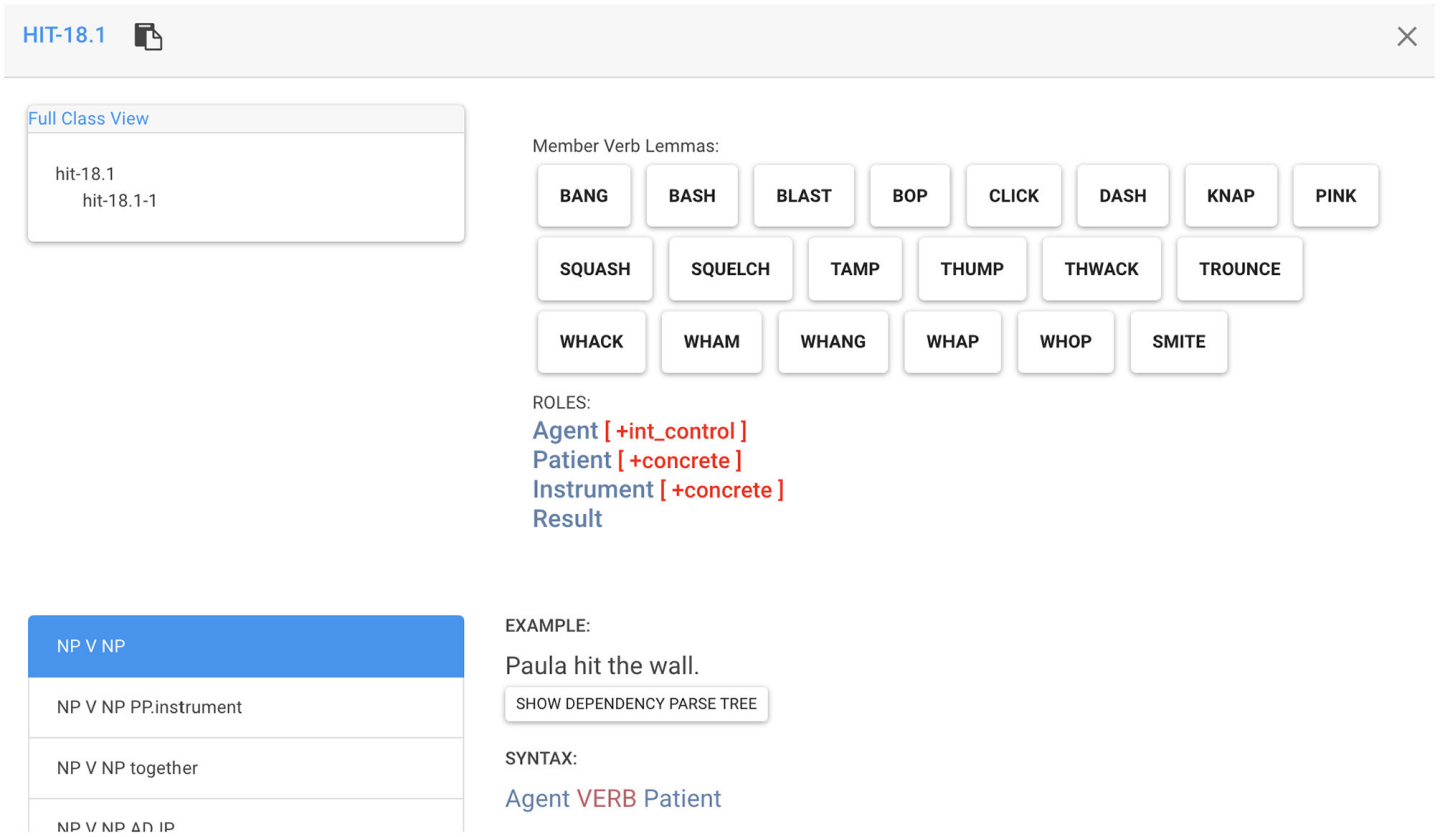
A screenshot of the VN *Hit-18.1* class. For a complete interactive view, see https://uvi.colorado.edu/verbnet/hit-18.1.

As an example, the VerbNet class *Hit-18.1* (Figure 2) demonstrates under ROLES that the verbs in this class have an AGENT tending to have intentional control (shown in the square brackets in front of the role), a PATIENT and an INSTRUMENT that tend to be concrete, and a RESULT. Under SYNTAX, we see the possible syntactic frames shared among the verbs in this class (including the grammatical relation for each constituent). Finally, under SEMANTICS, we see the semantic representations in the form of logical predicates.

The main difference between VerbNet and other semantic role providing lexical resources, such as FrameNet (Baker et al., 1998) and PropBank (Kingsbury and Palmer, 2002; Palmer et al., 2005), is that they lack the predicate logic-based semantic representations provided by VerbNet. VerbNet's explicit subevent sequences allow the extraction of preconditions and postconditions for many of the verbs in the resource and the tracking of any changes to participants. In addition, VerbNet abstracts away from individual verbs to more general categories of eventualities. Providing an integration of semantic roles, syntactic patterns, and first-order-logic representations for wide-coverage classes of verbs renders VerbNet a unique lexical resource, enabling a range of possible applications.

In recent years, we have revised VerbNet's semantic representations to make them more flexible and informative, including ways to incorporate verb-specific features into the general, class-level representations. We structured our representations following the well-defined theoretical framework of the Generative Lexicon (GL) (Pustejovsky, 1998). Classic GL characterizes the different Aktionsarten in terms of structured subevents, with states represented with a simple *e*, processes as a sequence of states characterizing values of some attribute, *e*_1_...*e*_*n*_, and transitions describing the opposition inherent in achievements and accomplishments. In subsequent work within GL, Pustejovsky (2013) integrated event structure with dynamic semantic models in order to more explicitly represent the attribute modified in the course of the event (the location of the moving entity, the extent of a created or destroyed entity, etc.) as a sequence of states related to time points or intervals. This Dynamic Event Model (Pustejovsky and Moszkowicz, 2011; Pustejovsky, 2013) explicitly labels the transitions that move an event from frame to frame.

Applying the Dynamic Event Model to VerbNet semantic representations allows a very precise tracking of participants across time and a nuanced representation of causation and action sequencing within a single event. We have introduced predicates that indicate temporal and causal relations between the subevents, such as **cause**(*e*_*i*_, *e*_*j*_) and **co-temporal**(*e*_*i*_, *e*_*j*_). We have also introduced variations on our standard thematic roles that indicate that more precise information can be inserted into the class-level semantic representations based on a specific verb's defined features. These roles are prefixed with V_, such as V_MANNER. For example, in the *Calibratible_cos-45.6.1* class, the semantic representation traces the change of the PATIENT along a scale, with V_DIRECTION as a place-holder for the direction given as a feature for a specific verb in the class. To see more details and examples of these changes, please see Brown et al. (2018), Brown et al. (2019), and Brown et al. (2022).

(1) *The price of oil rose by 500% from $5 to $25*.

**has_val**(*e*_1_, Patient, Initial_State)

**change_value**(*e*_2_, V_DIRECTION, Extent, Attribute, Patient)

**has_val**(*e*_3_, Patient, Result)

Members of this class have the feature values increase (e.g., “rise”), decrease (e.g., “fall”), or fluctuate (e.g., “vary”), which can replace V_DIRECTION when the representation is instantiated with a particular verb.

**Figure 3:**
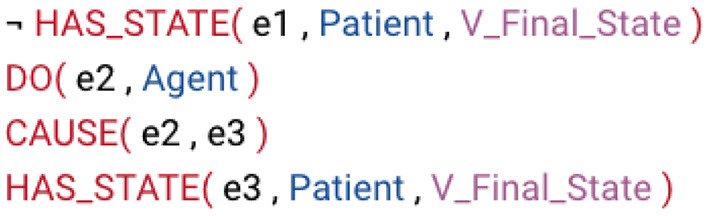
Semantics representations of the *Carry-11.4* class.

For demonstration purposes, we show the most prominent class that can be made more useful through subdivision into smaller subclasses: *Other_cos-45.4*. This class, along with its one subclass, cumulatively contain 303 verb members. VerbNet subclasses are hierarchical and inherit all the thematic roles, syntactic frames, and semantic representations from all the parent classes. For *Other_cos-45.4*, the thematic roles along with their selectional preferences include an AGENT [+int_control]^2^, a PATIENT, an INSTRUMENT, and a RESULT. Figure 3 displays the semantic representations for the syntactic frame *NP V NP* in the parent class, which corresponds to *Agent VERB Patient* syntactic constituent-semantic role mapping. The example sentence VerbNet provides for this syntactic frame is “Bill dried the clothes”. According to the provided syntax and semantics, “Bill” is the subject noun phrase and an AGENT, “dried” is the verb, and “the clothes” is the object noun phrase and a PATIENT. According to the semantic representation for this syntactic frame, the AGENT does something that causes the PATIENT to change from not having a particular state to having that particular state.

An interesting verb-specific component found for this class (see Section 3.3) was the property changed in the object. Feature values for this component included properties such as temperature, size, color, price, intensity, physical constitution, etc. The other ubiquitous semantic component was the direction of change, with the main feature values of decrease and increase. The next semantic component was result, with the main feature values being +improved, +damaged, +enabled, -joined, -hydrated, +joined, -improved, and +hydrated.

### 2.2. Dictionaries

We used web scraping on https://www.thefreedictionary.com/ to collect definitions for each verb lemma, limiting the output by part of speech. However, word sense disambiguation was performed manually among the remaining senses, selecting the sense closest to the verb sense in a given VN class. Sense definitions normally provide components of meaning, and that is what we harnessed to acquire verb specific features.

### 2.3. NLTK

NLTK (Natural Language Toolkit) is a suite of Python libraries and programs for symbolic and statistical NLP for the English language. Sentiment analysis is among the text classifiers provided by NLTK. In particular, it is a pretrained classification model, which is trained to classify text chunks into positive or negative sentiment predictions. Like any statistical language model, it returns the most accurate results when the input text is a chunk of text (i.e., a sentence or paragraph), not a token alone. The only chance that a verb alone can be correctly classified with regards to its sentiment is if the semantics of that verb has played a significant role in the learned weights of the model, and that is something we cannot tell. Despite that, we decided to trust the model to correctly predict the sentiment, at least for the verbs with a clear positive or negative sentiment component, such as “abhor”.

### 2.4. Brandeis Semantic Ontology (BSO)

The Brandeis Semantic Ontology (BSO) is a large Generative Lexicon (GL) ontology and lexical database which was designed to allow for more widespread access to GL-based lexical resources (Pustejovsky et al., 2006).

An essential characteristic of GL is its ability to capture the various dimensions of word meaning in a systematic manner. The basic vocabulary in BSO (Pustejovsky, 2001; Havasi et al., 2009; McDonald et al., 2018) and SIMPLE (Lenci et al., 2000; Busa et al., 2001; Lenci, 2001) relies on an extension of Qualia Structure (Pustejovsky and Bouillon, 1995) for structuring the semantic/conceptual types as a representational device for expressing multi-dimensional, orthogonal aspects of word meaning. Qualia Structure involves four different roles, that address different dimensions concerning the properties of a lexical item's meaning. The SIMPLE type system was the first attempt to provide a full ontology associated with GL-inspired analysis, integrating: a considerable enhancement of Qualia Structure; an explicit reference to event types and Aktionsarten; and aspects of the upper model from EuroWordNet, differentiated with Qualia roles (Vossen, 1998).

**Figure 4:**
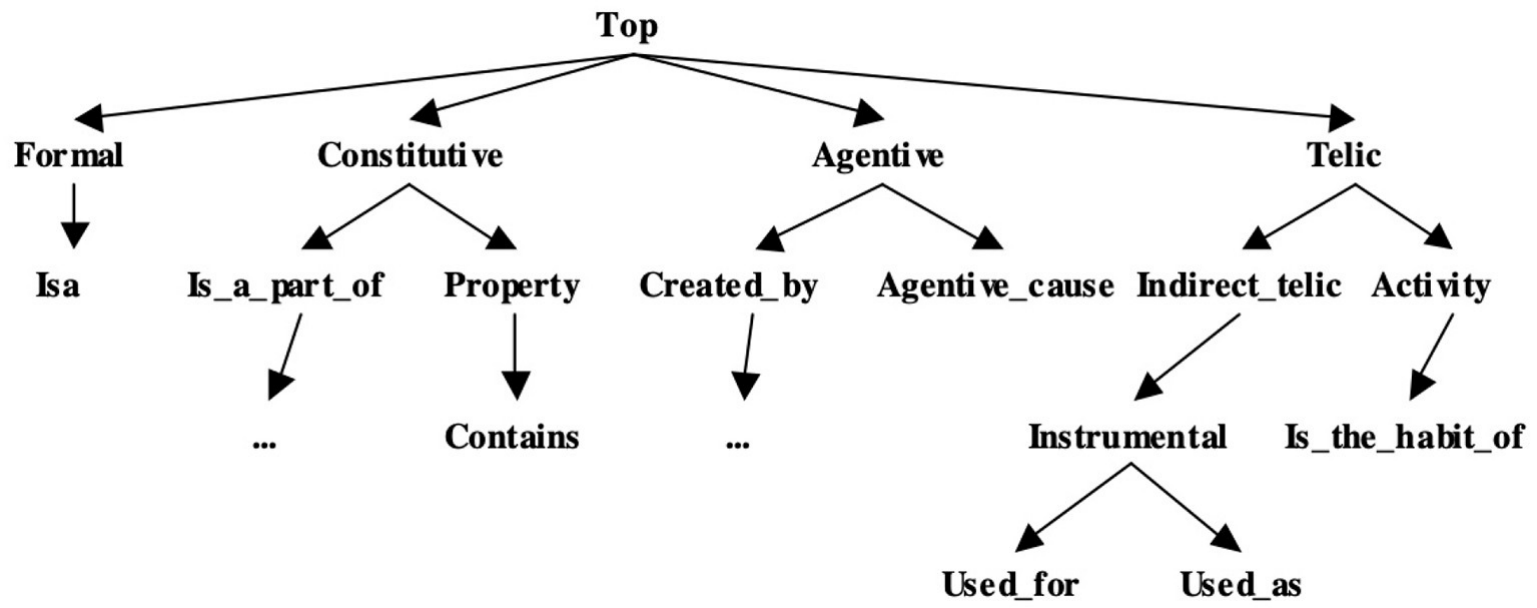
The extended qualia structure.

Thus, Qualia structure in SIMPLE and the BSO has been used as the basic syntax for constructing word meanings and each role can be viewed as an independent element or dimension of the vocabulary for semantic description. The possible values for the Qualia roles have been extended in BSO in order to express fine-grained distinctions between the large variety of semantic types, and the notion of Extended Qualia Structure has been introduced. Each of the four Qualia roles is the top of a hierarchy of other more specific Qualia information (formally expressed as relations between word senses or as features), representing more fine-grained subtypes of a given Quale which are consistent with its interpretation (see Figure 4 below).

BSO and SIMPLE sort out the various types of information entering into the characterization of a given word sense. Moreover, each piece of semantic information is also typed and inserted into structured hierarchies, each explicitly characterizing a certain aspect of the semantic content of nouns, verbs and adjectives. This way, the semantic information identifying word senses is fully explicit, and can directly and selectively be targeted by NLP applications. Finally, lexical information can be structured in terms of small, local semantic networks, which operate in combination with feature-based information and a rich description of the argument structure and selectional preferences of predicative entries.

BSO consists of 4,568 types, covering entities, events, and properties. BSO types are positioned in a hierarchical structure, allowing inheritance. Each BSO type [except the root node(s)] has a list of parents, therefore allowing multiple inheritance. Each BSO type has a set of entries, which consist of all the words/phrases subsumed by that BSO type, all of which share the set of qualia relations defined at the type level, but entries may have different parts of speech. For instance, the BSO type “Absorbent Substance”, with the parent “Functional Material”, has 13 entries, with some entries being an AdjectiveEntry (e.g., “absorbent”), some being a CollocationalNounEntry (e.g., ‘absorbent material'), and some being a NounEntry (e.g., “absorber”, “gauze”). BSO entry tags contain adjective, adverb, noun, verb, determiner, preposition, and their phrasal/collocational versions, as well as identifier (e.g., “inc”, “ltd”, “corp”), number (e.g., “eleven”, “zero”), ordinal (e.g., “eighteenth”, “first”), and title (e.g., “dr”, “st”).

## 3. Methods

In order to extract verb-specific semantic features, we resorted to several methods which grew into more accurate and more automatic methods as we progressed. In this section, we will provide an overview of each method used as well as all the resources that contributed to each method.

Since English is a language which frequently lexicalizes a manner semantic component in its verb inventory, it is important for any inference task to be able to abstract away from the manner component and, as a result, classify verbs into simpler events. For example, as illustrated in **Table 4**, 67 verbs in the *Run-51.3.2* class have a Manner of Motion semantic component. Abstracting away from that, there are 53 verbs in this class that could be subdivided into 5 simpler event types based on the following feature values: Traveling (e.g., “hitchhike”), Walking (e.g., “saunter”), Hiking (e.g., “tramp”), Sports (e.g., “bowl”), and Recreational Activities (e.g., “gambol”). Note that Sports is a subtype of and therefore inherits from Recreational Activities in BSO, where Sports is limited to Recreational Activities that are considered to be sports, such as Soccer, Basketball, or Skate. Also, there are 12 verbs in this class that contain an implicit direction of motion semantic component, and 12 verbs that contain a fast marker (indicating high speed of motion).

The results have been promising (see Section 4 for examples of some VerbNet classes and how BSO has helped in further decomposing the verbs in these classes). The results and their potential implications will be discussed in Sections 4 and 5.

### 3.1. Introspection and Dictionary Lookup

Our first approach to this task resorted to introspection and dictionary lookup described in 2.2. We analyzed five classes using this time-tested method (including *Run-51.3.2, Push-12, Calibratable_cos-45.6.1, Caused_calibratable_cos-45.6.2*, and *Roll-51.3.1*) before turning to more innovative approaches.

### 3.2. Sentiment Features From NLTK

Recourse to this method originated from the observation that VerbNet class members enjoy similar semantic and syntactic patterns, and by the same token, each class contains a limited number of thematic roles to which the arguments (participants) of these events (verbs) in that class can correspond. Among these, there are classes with EXPERIENCER and STIMULUS thematic roles, which, according to the role definitions usually indicate cognitive events, but also physical events causing some type of feeling in the EXPERIENCER. An EXPERIENCER is defined in VerbNet as a patient that is aware of the event undergone, which often involves an emotional or psychological response elicited by a Stimulus (specific to events of perception), while a STIMULUS is defined as a cause in an event that elicits an emotional or psychological response (specific to events of perception).

Consequently, these VerbNet classes are semantically about an external STIMULUS creating a certain cognitive state in a human EXPERIENCER. As a result, we decided to use the automatic sentiment analysis library from the Natural Language Toolkit (NLTK) (Loper and Bird, 2002) (described in 2.3) to automatically label each verb in a given VerbNet class. Essentially, we asked the sentiment analyzer to classify the verb members of any given VerbNet class into three subclasses. To that end, verb lemmas were fed into the sentiment analyzer in isolation, in hopes that it would assign a sentiment label to each verb lemma based on the data on which it is trained. We assumed that these three subclasses would represent positive, negative, and neutral feelings. The VerbNet classes we subdivided using this method were *Admire-31.2* and *Marvel-31.3*. This method produced useful results but was abandoned in favor of the BSO method, described below, which proved more accurate, as discussed in Section 4.1.

### 3.3. BSO Mapping

The Brandeis Semantic Ontology (BSO) was introduced in Section 2.4. The potential benefit of using BSO to extract verb semantic features in VerbNet classes first became apparent when we were mapping between VerbNet and BSO. Scrutinizing the BSO types led us to suspect that BSO might be an especially suitable resource for identifying verb-specific semantic features. Among BSO types that confirmed this hypothesis were “Decrease Temperature” (e.g., “chill”), “Increase Temperature” (e.g., “heat”), “Decrease Size” (e.g., “lessen”), “Increase Size” (e.g., “lengthen”), “Decrease Intensity” (e.g., “attenuate”), “Increase Intensity” (e.g., “amplify”), “Cause Privative Constitutive” (e.g., “disintegration”), “Turn Into Mass Substance” (e.g., “pulverize”), and “Change of Physical State” (e.g., “ripen”), to name but a few, which are all extremely useful event types for subdividing the huge (340 members) *Other_cos-45.4* class.

These informative distinctions were not restricted to change of state events. For instance, BSO types also helped subdivide the *Sight-30.2* class into five subclasses, analogous to the five human senses minus the touch sense (Hear, See, Smell, Taste), and one general perception type (Perception). Examples included “See Activity” (e.g., “behold”), “Hear Activity” (e.g., “overhear”), “Smell Activity” (e.g., “sniff”), “Taste Activity” (e.g., “savor”), and “Perception Activity” (e.g., “experience”).

We used the following method. First, for every verb member of a given VerbNet class, we collected all the BSO types that had that verb as an entry, as well as the parents of those types. Then, for each of these BSO types, we counted the number of verb entries they had in common with the VerbNet class in focus, and sorted the BSO types based on that. The more verb lemmas a BSO Type and a VerbNet class had in common, the more likely that a (verb lemma, VerbNet class) tuple would map to that BSO type, and therefore that BSO type has more potential for suggesting the verb-specific semantic features for that (verb lemma, VerbNet class), and would be more highly ranked. Finally, we asked a human judge familiar with VerbNet to choose, for every (verb lemma, VerbNet class) pair, the best BSO types to represent them, taking into account that the best types would likely (but not necessarily) be at the top of the list.

So far, we have extracted verb-specific semantic features for 25 VerbNet classes using this method, with additional classes still being examined. It should be noted that many VerbNet classes are too small to allow subdivision. The results are presented in the Section 4 and discussed in the Section 5.

## 4. Results

VerbNet has 328 classes. We consider every class with all its subclasses^3^. For example, the *Carry-11.4* class (with 15 verb members) has two subclasses: *carry-11.4-1* and *Carry-11.4-1-1* (with 5 verb members), together making 20 verb members.

**Figure 5:**
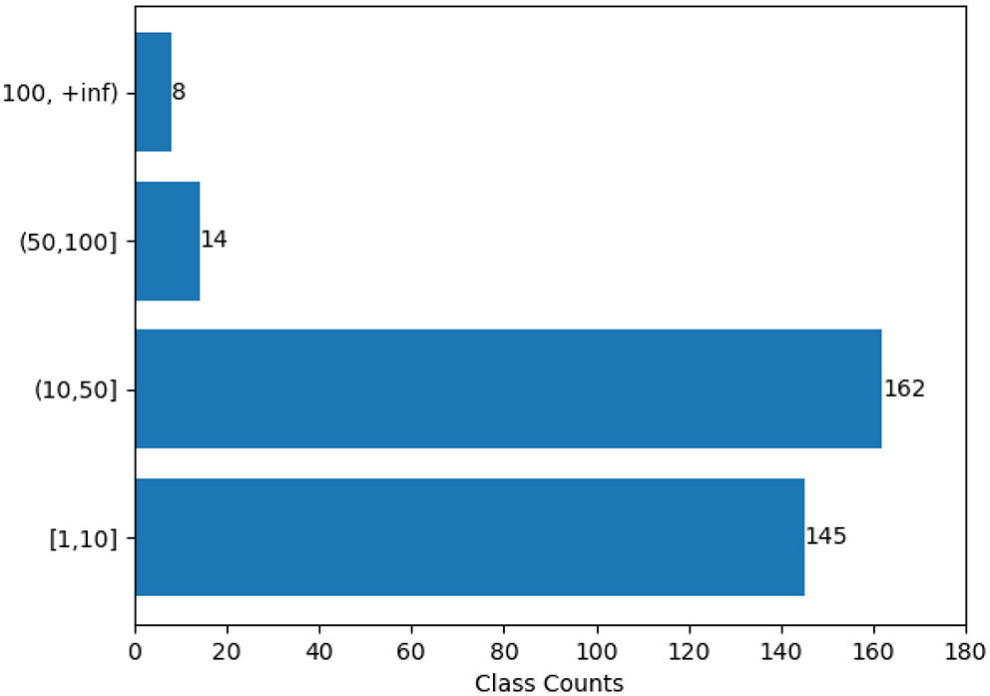
Distribution of VerbNet classes based on their verb member count.

There are currently 4,559 unique verbs in VerbNet, and 6,745 verb_member-verb_class pairs. Among these 329 classes, there are 184 classes with more than 10 verb members as seen in Figure 5. On the other hand, BSO contains 4,936 unique verbs, and 6,078 verb-BSO_Type pairs. There are 1,905 verb entries in BSO that are absent from VerbNet. This section is restricted to a brief overview of the number and type of classes handled with different methods. The coverage of Method 1 (Section 3.1) was limited and has already been discussed so this section will focus on Methods 2 (Section 3.2) in 4.1 and 3 (Section 3.3) in 4.2. Section 5 discusses the pros and cons of the methods in more depth.

### 4.1. Sentiment Analysis

For classes involving positive and negative sentiment (*Amuse-31.1, Admire-31.2, Marvel-31.3*, and *Nonverbal_expression-40.2*), we used the NLTK sentiment classifier to tag the verbs automatically. We then asked a human annotator to perform a one-time post-classification annotation to judge whether the labels were correct. Table 2 shows the precision and recall on the task. We will discuss these results in Section 5.

**Table 2:** Using NLTK sentiment analysis on the verb members of a few VerbNet classes.

	**Positive (%)**	**Negative (%)**	**Neutral (%)**
Precision	96.83	100	27.97
Recall	51.69	50.87	97.06

We also assessed the correspondence between NLTK sentiment analysis and BSO types indicating some type of sentiment, for the four VerbNet classes we ran the sentiment analyzer on. Since BSO is a manually curated resource, and NLTK sentiment analyzer is trained on substantial amounts of manually tagged training data in context, we compared NLTK to BSO. For the verbs in these four VerbNet classes that were automatically classified as one of the positive, negative, and neutral labels, we aggregated the BSO-derived verb specific features into these three sentiment classes. For example, (Mental Attitude: Appreciate) was considered a positive label (Mental Attitude: Dislike) as negative, and (experiencer cognitive state: +attention) as neutral. Given these considerations, we evaluated the performance of the sentiment analyzer against the BSO-derived labels. Table 3 illustrates the results. Note that the precision for positive and negative classes is high, indicating substantial agreement, but there is significant disagreement about neutral judgements. Examples of verbs clearly mislabeled by NLTK as neutral include detest, grudge, and deplore which should be negative, and esteem, relish, and revere which should be positive (and they are of course correctly classified based on BSO).

**Table 3:** Evaluating the performance of NLTK sentiment analyzer on the sentiment of four classes against the labels derived from BSO.

	**Positive**	**Negative**	**Neutral**	**Global**
Precision	0.82	0.92	0.26	0.59
Recall	0.56	0.59	0.66	0.59
Macro average F1 score				0.63

### 4.2. BSO Mapping

As mentioned in Section 3, the most promising method was using the mapping between VerbNet and BSO. The intersection between the set of verbs in the two resources contains 3,023 verbs, covering 66.02% of VerbNet and 61.24% of BSO. For each VerbNet class, we found a number of shared BSO types, usually from the set of parent types, which indicated some sort of semantic component shared among most members of the class. For example, almost all verbs in the *Amuse-31.1* class mapped to the BSO type “Cause Cognitive State”, which makes it amenable to a sentiment analysis method due to the reason described in 3.2, but a BSO analysis proved even more useful.

The information contained in BSO types *name* field were of a substantial assistance in detecting categories of physical causality introduced by Gao et al. (2016) (see Table 1). For example, the BSO type “Cause Decrease Size” in terms of physical causality categories indicates a change in Dimension in the attribute Size with the attribute value decreases. Or, the BSO type “Privative Contain Relation” indicates a change in physical property in the attribute containment with the attribute value emptied. The verb “slice” in the pizza slicing example from Gao et al. (2016) maps to the “Separate Activity” BSO type (along with 81 other verbs). We will now take a look at the informative physical causality categories we were able to extract from BSO.

#### 4.2.1. Extra Large Classes

**Figure 6:**
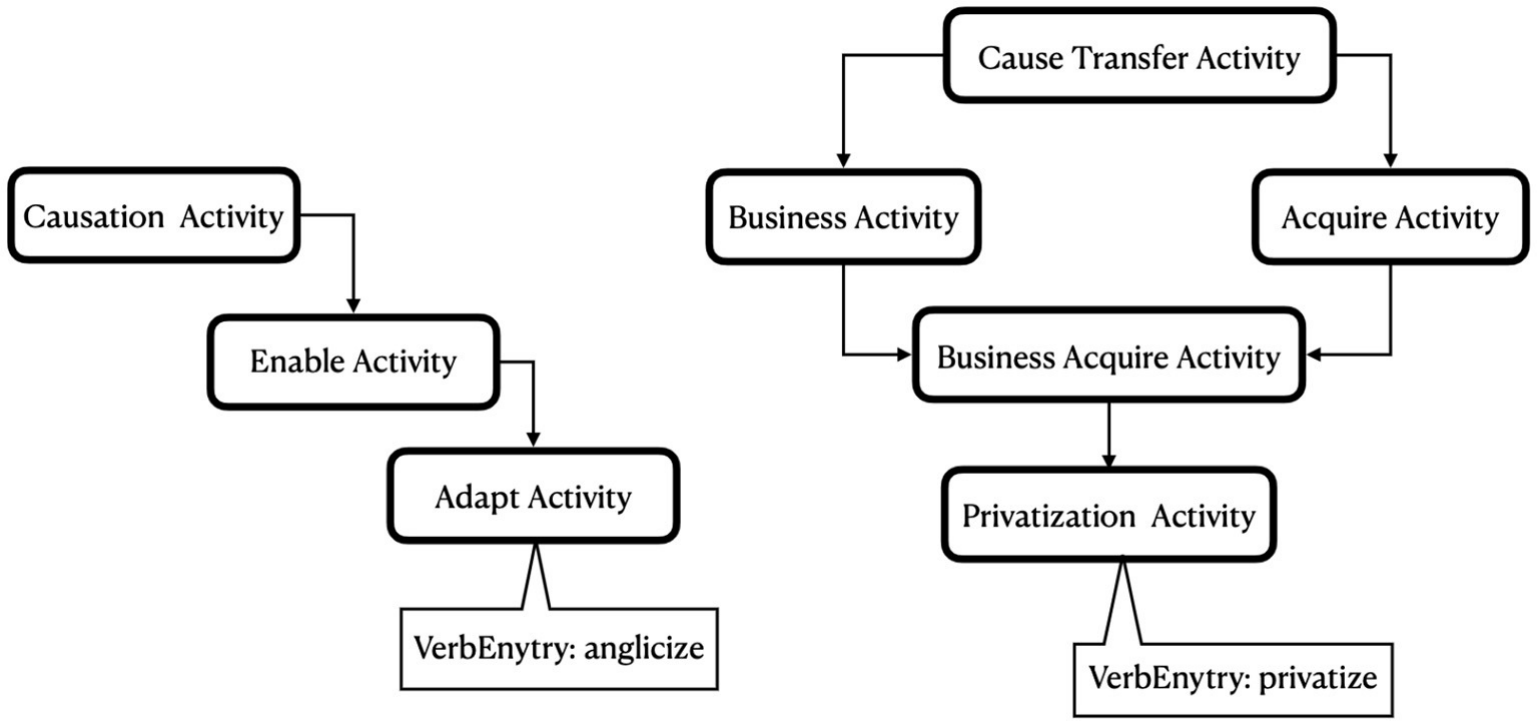
Example of hierarchy in BSO types that illustrates distinct mappings to two members of the *Other_cos-45.4* class: “anglicize” and “privatize”.

**Figure 7:**
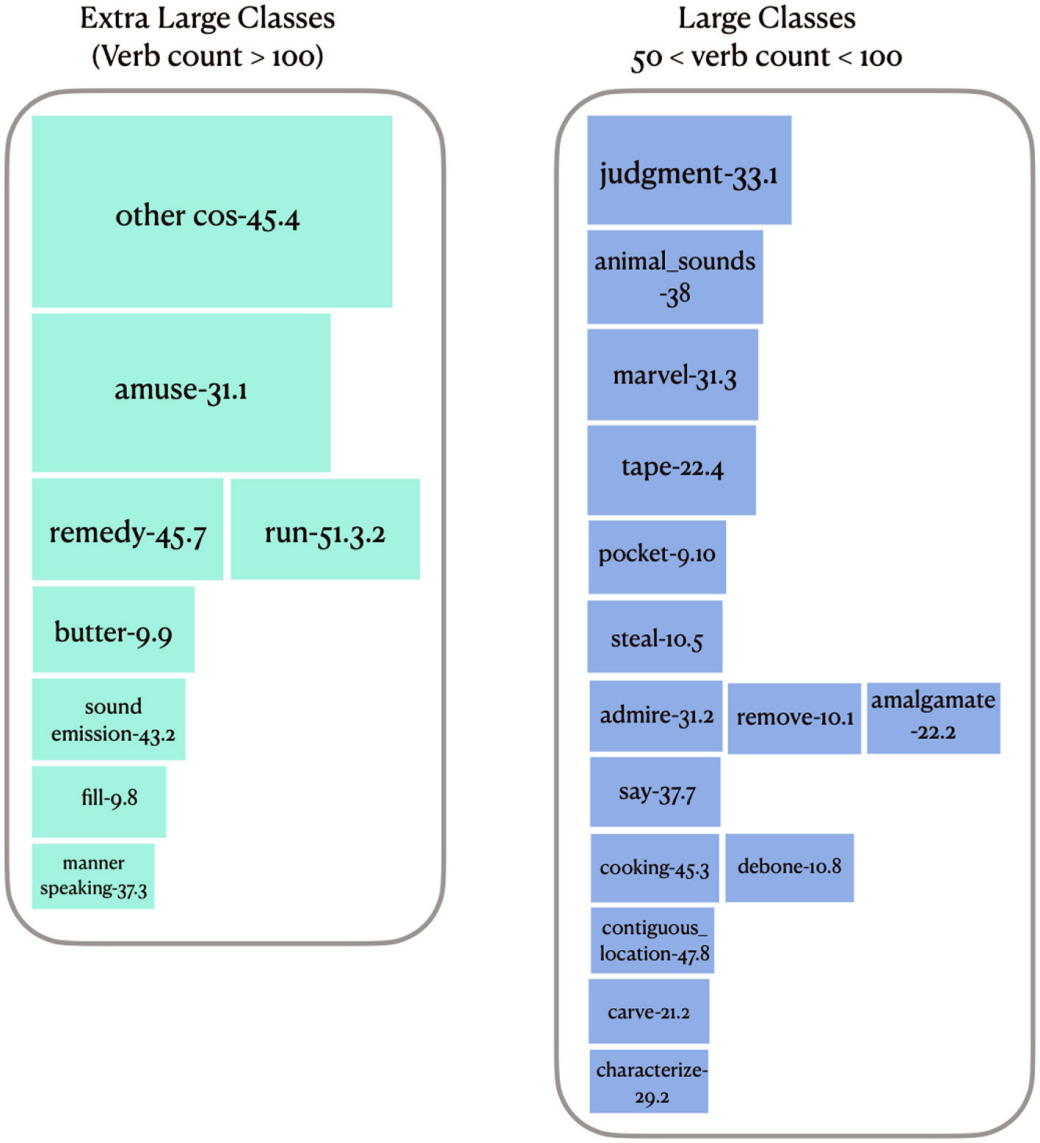
VN classes with extra large (verb member count more than 100) and large sizes (verb member count between 50 and 100). The width of each container is equal to the size of the class in the extra large classes, and scaled to twice the size of the class in large classes.

VerbNet has 8 classes with more than 100 verb members, with 1,349 verbs, including 1,263 unique verbs (see Figure 7). These are the main target classes benefiting from identifying verb-specific semantic components. Each (verb lemma, VerbNet class) pair may have zero or more verb-specific features. For example, the verb “chill” in the *Other_cos-45.4* class has the following two features: property changed: temperature; direction of change: decrease. These were extracted from the BSO type “Cause Decrease Temperature”. The same verb, in its other sense, maps to “Cause Negative Feeling” and “Fear Activity” BSO types, which are the mappings for this verb when it occurs as a member of the *Amuse-31.1* class, resulting in two types of end cognitive state: negative feeling and fear. Note that the categories of physical causality suggested by Gao et al. (Table 1) are a comprehensive yet non-exhaustive set of change types that are limited to physical changes. Hence, cognitive changes or any other abstract changes are not represented in Table 1. An example of an abstract change in the *Other_cos-45.4* class is “anglicize”, which is represented by the BSO type “Adapt Activity”, which in turn inherits from “Enable Activity” (1 step remove) and “Causation Activity” (2 steps remove), but there is no BSO type indicating an abstract change. In fact, all abstract changes are represented by other BSO types. For example, the verb “privatize” in the same VerbNet class maps to the BSO type “Privatization Activity”, which inherits from ‘Business Acquire Activity' (1 step remove), “Acquire Activity” (2 steps remove), “Business Activity” (2 steps remove), and “Cause Transfer Activity” (3 steps remove) (see Figure 6). There is also a general lack of verbs indicating chemical processes in BSO, such as “alkalize” or “carbonize”. Table 4 illustrates the set of subdividing semantic features for five of these extra large classes, along with their values found from BSO types.

**Table 4:** Five of the VerbNet classes with more than 100 members.

Other_cos-45.4	property_changed (139)	physical state (20), intensity (18), size (16), color (12), physical constitution (11), existence (8), quality (7), temperature (7), speed (6), strength (3), taste (3)
Amuse_31.1	end_cognitive_state (205)	negative feeling (122), positive feeling (49), surprise (15), fear (8), +attention (6), -attention (3)
Remedy-45.7	result (39)	+improve (11), -enabled (7), +created (3), +enabled (3), +damaged (3), -communication (3)
run-51.3.2	manner_of_motion (67)	
butter-9.9	covering_entity_type (65)	Functional Material (12), Clothing Artifact (12), Substance (11), Drugs and Medicine (7), Solid Substance (4), Combustible (3), Representational Artifact (3)

*Values occurring less than three times in a class have been disregarded in the table for brevity. Also, only the semantic features with more than 10 verbs have been included in the table, for the same reason*.

Overall, the verbs in these classes have a 61.44% overlap with BSO verbs. Examples of VN verbs which are absent from BSO include “Americanize”, “alkalize”, “alkalify”, “bolshevize”, “carbonize”, “crystalize”, “glutenize”, “resuscitate”, etc.

#### 4.2.2. Large Classes

**Figure 8:**
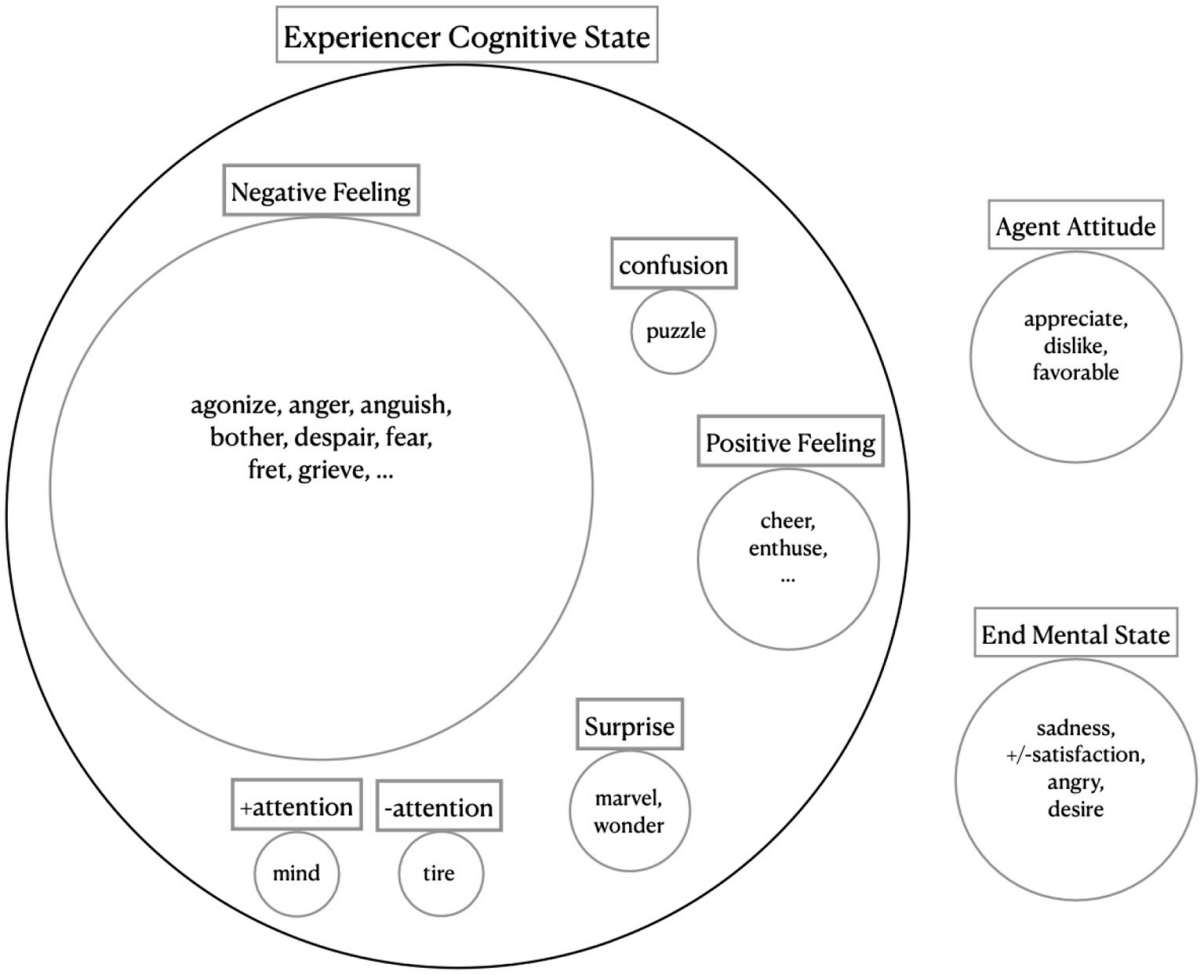
A summary of the verb specific semantic features in the *Marvel-31.3* VN class.

There are 14 classes having between 51 and 100 members (852 verbs in total, including 827 unique verbs) (see Figure 7). 66.75% of these verbs overlap with BSO. For example, the verb “marvel” in the *Marvel-31.3* class has the component experiencer cognitive state: surprise, which was extracted from the BSO type “Cause Surprise Activity” (having “Cause Cognitive State” as its parent). experiencer cognitive state was the main subdividing feature in this class (28 verbs having 31 values), with values such as negative feeling, positive feeling, fear, surprise, ±attention, and confusion. But there were also 8 verbs containing a type of agent attitude component, and 10 containing a type of end mental state (see Figure 8). Out of 72 verbs in this class, BSO types provided us with semantic components for 42.

The verbs in the *Judgment-33.1* class are mainly subdivided based on the type of agent attitude (including favorable, negative, +respect, -respect, and forgive), and event type (e.g., opposition, celebrate). No distinctive features were found in BSO for the verbs in *Animal_sounds-38* class. Table 5 illustrates the set of subdividing semantic features for five of these classes with 50 to 100 verb members, along with their values found directly from BSO types.

**Table 5:** Five of the VerbNet classes having between 51 and 100 members.

**VerbNet Class**	**Semantic Components**	**Values**
judgment-33.1	agent_attitude (52)	negative (20), favorable (17), -respect (9), forgive (3)
marvel-31.3	experiencer_cognitive_state (31)	negative feeling (18), positive feeling (6)
pocket-9.10	container_type (35)	architectural object (9), container (7), household artifact (6), material object with instrument telic (3)
steal-10.5	activity (31)	acquire (14), capture (5), steal (5)
say-37.7	speech_act_type (39)	reporting (13), say (12), imply (4), advice (4)

*Values occurring less than three times in a class have been disregarded in the table for brevity. Also, only the semantic features with more than 10 verbs have been included in the table, for the same reason*.

#### 4.2.3. Medium Classes

There are 162 VN classes having between 11 and 50 members (3,661 verbs in total, including 2,667 unique ones). 70.49% of these verbs overlap with BSO. In the *Destroy-44* class, for instance, the verb “annihilate” has an end state of destroyed for the patient, while the verb “efface” has an end state of removed, and the verb “shatter” has an end state of broken. In the *Disassemble-23.3* class, the verbs “unscrew” and “unlock” both have result: open as a semantic component. Naturally, the classes with this scale contain fewer semantic features compared to larger classes. For example, the *Breathe-40.1.2* (22 verbs) class yields no further semantic component (see Table 6).

**Table 6:** Five of the VerbNet classes having between 11 to 50 members.

**VerbNet Class**	**Semantic Components**	**Values**
spank-18.3	instrument_type (11)	Clothing Artifact (3), Weapon (3), Sport Artifact (2), Body Part (2), Wood (1)
destroy-44	end_state (19)	Destroyed (12), Removed (2), Disease (1), Defeat (1), Damaged (1), Nonwholeness (1), Broken (1)
knead-26.5	process (6)	Hit (2), Amass (1), Change of Physical Constitution (1), Move (1), Force (1)
entity_specific modes_being-47.2	end_state (5)	specific shape (3), specific end product (2)
breathe-40.1.2	-	-

#### 4.2.4. Verb Particle Constructions

Verb Particle Constructions (VPCs) in English are a rich source of multi-word expressions (MWEs). VPCs combine a head verb with one or more obligatory particles, in the form of prepositions (e.g., “figure out”, “give in”), adjectives (e.g., “come short”, “let alone”) or verbs (e.g., “let go”^4^). VPCs with prepositional particles are the largest subset (Bannard, 2005; Cook and Stevenson, 2006).

Both VerbNet and BSO contain MWEs in the form of VPCs. In VerbNet, VPCs are treated as atomic non-compositional verbs. Verb members that are VPCs are represented with the whitespace substituted by an underscore. An example is the VPC “go_on” in the *Begin-55.1* class, or the VPC “figure_out” in the *Discover-84-1* class.

On the other hand, BSO tags VPCs as *CollocationalVerbEntry*, keeping the whitespace between the pieces. For example, the VPC “hand down” belongs to the BSO type “Give Activity”, and the VPC “pass away” belongs to “Go Out of Existence”.

The VPC “go on” in the *Begin-55.1* class is mapped to the BSO type “Continue Activity”. The *Begin-55.1* class in VerbNet is an aspectual class, marking the beginning of the occurrence of an event or a participant's engagement in an event. BSO's “Continue Activity” and “Begin Activity” pinpoint the distinctions between different verbs in this class. Interestingly, both “Continue Activity” and “Begin Activity” inherit from the “Aspectual” BSO type, which supports the VerbNet class membership of these verbs. Another BSO type inheriting from “Aspectual” is “End Activity”, which is represented in the *Stop-55.4* class. This class has the same semantic representations as the *Begin-55.1* class, only with reverse ordering of the subevents [¬**occur**→**occur** (*Begin-55.1*) vs. **occur** → ¬**occur** (*Stop-55.4*)].

### 4.3. Dissemination

In order to make this additional layer of information publicly available, we added the verb-specific semantic features to the representation of verb members in the VerbNet class XML files. In the XML files, each verb member has a series of attributes, including mappings to other resources such as WordNet, FrameNet, PropBank and OntoNotes Groupings. We added verb-specific semantic features under the attribute *features*. We provide further details below.

#### 4.3.1. Unified Verb Index—Revamped

**Figure 9:**
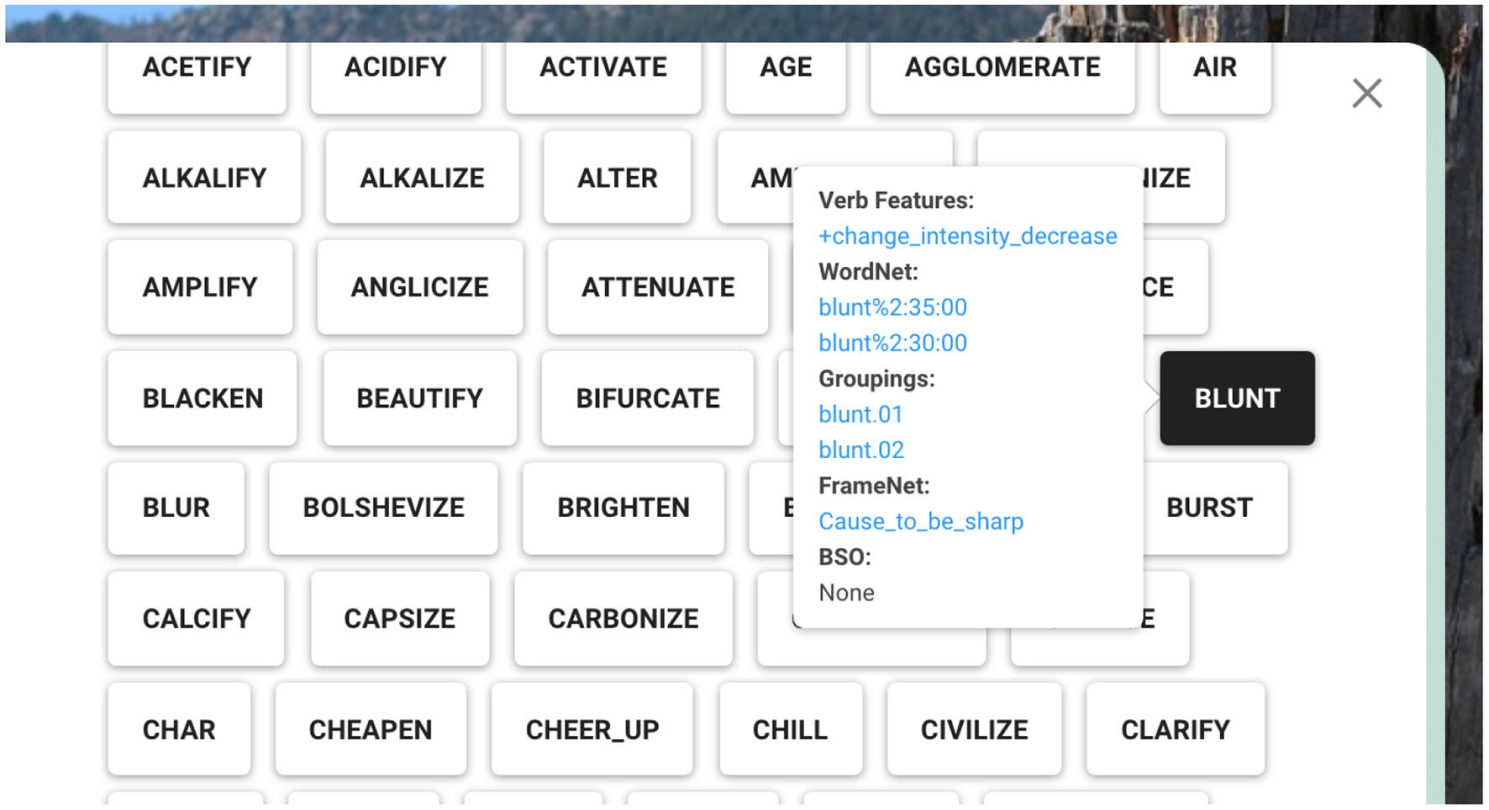
Verb-specific features popping up as a result of hovering over the verb “blunt”.

As a user interface for multiple lexical resources, the Unified Verb Index (UVI) has been developed and expanded over the years (as shown in Figure 9). More recently, with the growth of the information layers in the VerbNet XML files, and considering that the old UVI was not capable of graphically presenting the new information, the need for a revamped UVI arose. This completely revamped UVI can be accessed at https://uvi.colorado.edu/.

The main functionality of the UVI is providing a search system able to search over VerbNet, PropBank, FrameNet, and OntoNotes Sense Groupings at the same time. The VerbNet part of the query result demonstrates the class containing the searched lemma. Hovering over each verb member pops up mapping information as well as the verb-specific semantic features introduced in this work. A screenshot is provided in Figure 9 for illustration purposes.

In addition to that, any information regarding the VerbNet project is either presented directly on the UVI, or a link to the relevant external pages can be found on the UVI website. For example, in the Reference Pages tab, we can find definitions (if available), class occurrences, and frequency of occurrence for the VerbNet Thematic Roles, Semantic Predicates, Selectional Restrictions on the arguments, and Syntactic Restrictions. Also on the same page, Preposition Class Hierarchy and Thematic Roles Hierarchy are illustrated.

On the Resources subtab of the NLP Applications tab, we find links to the VerbNet API GitHub repository, SemLink GitHub repository (Palmer, 2009; Bonial et al., 2013; Stowe et al., 2021), the recently published VerbNet semantic parser (Gung, 2020) GitHub repository as well as a demo page. There is also information about some external NLP projects that have used VerbNet. In the Download subtab, the VerbNet XML files (in any version of choice) can be downloaded in json format.

#### 4.3.2. Verb-Specific Features

**Figure 10:**
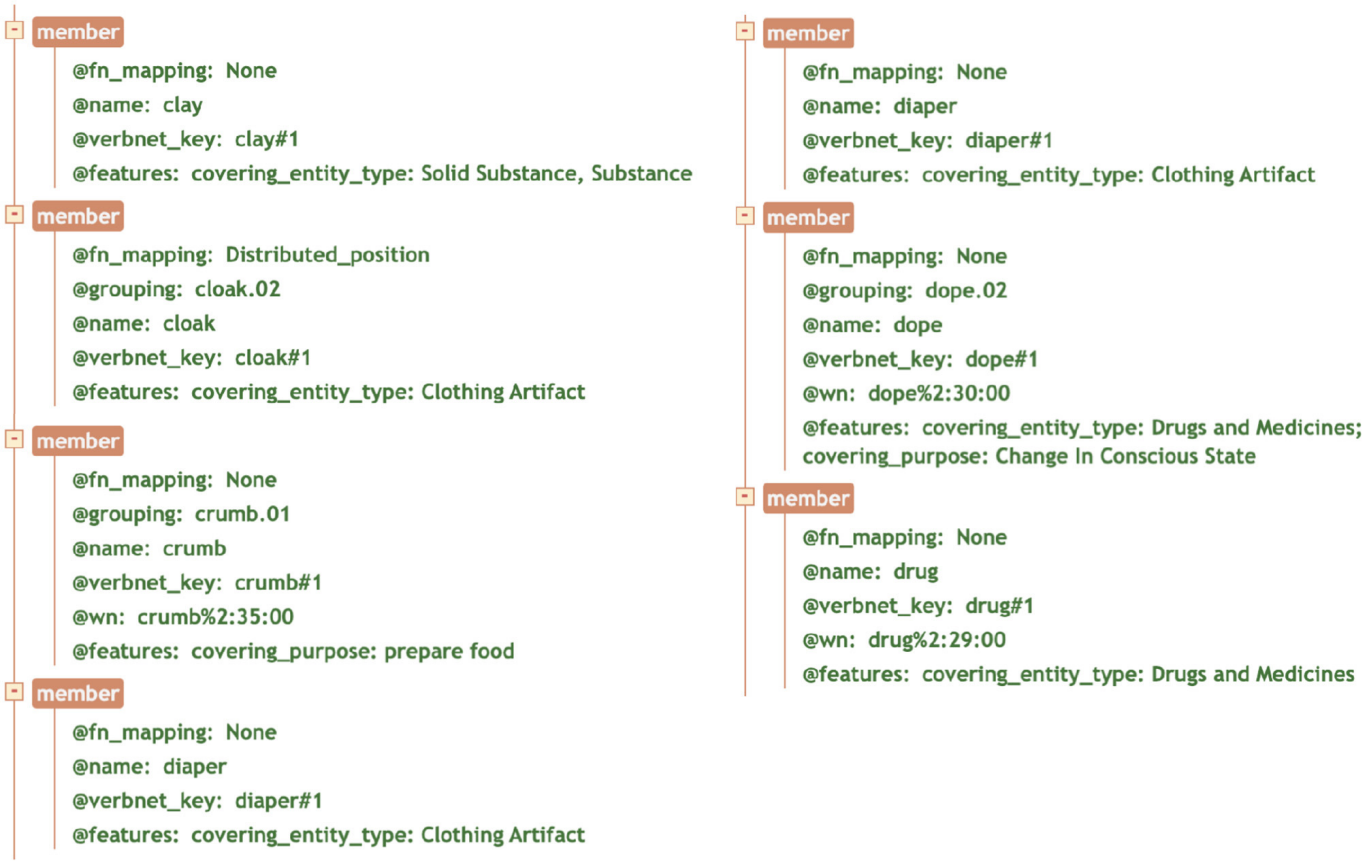
A snapshot of the tree view of the XML file for the *Butter-9.9* class, selected verbs that contain verb-specific features. @fn_mapping = FrameNet mapping; @grouping = OntoNotes grouping; @name = verb lemma; @verbnet_key = sense disambiguation of the verb in verbnet; @wn = WordNet mapping; @features = verb-specific features, as a list of key-value pairs (feature type: feature value).

The verb-specific features extracted from BSO were integrated into the UVI. Since each verb-specific feature specifies the semantic components of a single verb, the integration was at the MEMBER level, which is the special element representing verb members according to the defined XML schema. Each of the attributes starting with @ in Figure 10 have been defined as an attribute of the MEMBER element, with *features* being optional, and the feature values being a list of acceptable values, as defined in the schema.

## 5. Discussion

One of the underlying reasons for starting this project was that an extra large class such as *Other_cos-45.4* has so far been an amorphous collection of verbs indicating some type of change of state. Some of the applications using VerbNet (Kozierok et al., 2021; Martin, 2021) could have benefited much more from this resource if it had included the recently added fine-grained verb-specific semantic features. A reasoning system would benefit from explicit information about the type of the property that is changing in an event, such as temperature (e.g., “cool”), speed (e.g., “accelerate”), color (e.g., “bleach”), or size (e.g., “dilate”), etc.

Since BSO is manually curated and the BSO types attributed to each verb-VerbNet_class pair were also manually selected among all possible BSO types for that pair, this method is highly precise. However, the recall is lower than desired due to the fact that many verbs existing in VerbNet do not exist in BSO (see Section 3.3). Also, for some of the polysemous verbs that are in both resources, there are some senses that exist in one resource and are absent from the other. These are part of the limitations of this method.

In addition to high precision, another advantage of using BSO is that it illuminates semantic features that we may have otherwise ignored. For example, comparing the results of the automatic sentiment analysis method (Section 3.2) and the BSO mapping method (Section 3.3), it might come as a revelation to an NLP researcher that human cognitive states are not limited to two or three types of feeling (positive, negative, neutral) as is commonly assumed in NLP sentiment analysis tasks. Rather, there are other types of cognitive states that we experience, such as attention or lack of attention, surprise, confusion, etc. A binary classification of cognitive states into positive or negative is quite simplistic and of limited use. Such insights are among the benefits we receive by using pre-existing manually-curated resources such as BSO. They also corroborate the findings of a Twitter emotion recognition task (Saravia et al., 2018), where the following emotion types were detected: sadness, joy, fear, anger, surprise, trust, disgust, and anticipation. The features provided in VerbNet can therefore be helpful in knowledge-aware labeling and training data generation for such classification tasks as well.

The *Judgment-33.1* class is another example of BSO being more fine-grained. In the introspection step adopted in the beginning, we had only assumed a simple polarity component for this class. The BSO types, however, led us to a much more detailed and complete picture of the verbs' semantic components in this class. Although polarity is still to be the main component (with 17 favorable and 10 negative), there are also 9 disrespect events (e.g., “ridicule”), as well as other more fine-grained, less frequent components.

Another important piece of information this process could shed light on is the direction of change semantic component. There are some classes that are expected to have this semantic component, as they indicate a change of state, such as *Other_cos-45.4* or *Caused_calibratable_cos-45.6.2*. However, we also found other less intuitive classes that contained this semantic component. For instance, in the *Remedy-45.7* class, direction of change is one of the main semantic features. This feature in particular is among the most useful features we obtain, since the direction of change has notoriously been misclassified in neural approaches (Yih et al., 2012) and is a critical piece of information in the downstream tasks where it matters. Recently developed transformer models such as BERT have significantly improved on identifying this distinction, but for domains with grounded tasks such as Human Robot Interaction where language understanding still relies on logical predicates and deterministic methods more than on distributional methods, the information contained in verb specific features could be crucially useful.

This feature is also observed in classes indicating some sort of motion, such as the *Run-51.3.2, Assuming_position-50*, or *Meander-47.7* classes. These include specific values such as descend, ascend, or fall, but could also indicate a general (non-specific) sense of direction that is important in the semantics of an event. An example for this is the verb “march” which indicates a directed motion, but the direction is unspecified.

Result states are another important element to examine which could be useful for an inference system. In the *Fill-9.8* class, for example, there are 14 verbs that indicate an improved appearance of the theme (e.g., “trim”), 5 that indicate a containment relation (e.g., “saturate”), and 4 that indicate a resulting dirty state (e.g., “stain”). The improved appearance as a resulting state also occurs in the *Butter-9.9* class (e.g., “glaze”). Any reasoning system requiring inference, particularly a system that needs to track the states of entities, would benefit from explicitly learning the end state of an event participant in dealing with natural language—something typically categorized as world knowledge. These explicit information elements can be fed into machine learning algorithms as extra layers of information to see whether the predictive power of a language model improves. Kazeminejad et al. (2021) use result states from the VerbNet semantic representations generated by the VerbNet semantic parser (Gung, 2020) to predict changes in entity states. Particularly, the arguments labeled as RESULT can be extracted as the consequent states when they are an argument of a stative primitive predicate such as **be** or **become**. Kazeminejad et al. (2021) introduced Lexis, a system for predicting entity states in procedural paragraphs. In the ProPara dataset (Clark et al., 2018; Mishra et al., 2018), which is the dataset Lexis is evaluated against, the types of tracked changes include changes in location or existence state of entities (i.e., whether they are created or destroyed at a certain step). However, these are not the only types of physical change that are desirable to be tracked. Other datasets, such as Recipes (Bosselut et al., 2017), track different types of state change, including LOCATION, COOKEDNESS, TEMPERATURE, SHAPE, CLEANLINESS, and COMPOSITION. Again, it is predicted that the layer of information proposed in this work can contribute to such entity state tracking systems as a symbolic utility. In fact, the second version of Lexis will use the verb-specific semantic components as an extra layer of information to pinpoint the types of state changes in entities, and this version will be evaluated on both the ProPara and Recipes datasets.

## 6. Conclusion and Future Work

In this paper, we have discussed the addition of verb-specific semantic features that allow subdivision of heterogeneous VerbNet classes into more coherent and informative subclasses. This additional information enhances VerbNet's utility as a resource for natural language inference, greatly increasing the range and specificity of possible inferences. In defining these features for VerbNet classes we found a pre-existing manually curated lexical resource, BSO, to be especially valuable. The carefully curated, fine-grained semantics of BSO was used automatically to suggest relevant verb-specific semantic features, which were then hand-checked. As noted above, for many verb classes, the syntactic classification that gives rise to Levin-style clustering of verbs does not differentiate based on semantic orientation or direction for change of state or scalar change verbs, an unfortunate limitation to VerbNet's usefulness (and FrameNet's). However, these distinguishing characteristics or feature values for such components are often identifiable in the related BSO type, and identification of the aligned verbs as a subclass in VerbNet can be achieved semi-automatically.

We are considering augmenting VerbNet in the future with BSO verb entries that VerbNet currently lacks. This process could be expedited by the BSO types that frequently map to verbs in a given VerbNet class. Of course the ultimate decisions regarding class-membership need to be made by linguistics experts, since VerbNet class membership depends on both semantics and syntax, whereas BSO only considers semantics. This feature of VerbNet also makes it amenable to using distributional semantics to automatically classify new verbs based on the distance of their word embeddings to the embeddings of existing verbs, especially for verbs that are not polysemous.

So far we have not found any significant conflict between Levin and BSO semantic characterizations. However, we plan to carry out a more theoretical exploration of Levin and BSO in the future.

## Data Availability Statement

Publicly available datasets were analyzed in this study. This data can be found here: https://uvi.colorado.edu.

## Author Contributions

The original suggestion for adding verb-specific mappings to the run class based on specific semantic features came from JP in a discussion with all of the authors. GK worked out the details of applying those features to that class and then moved on to other classes, in frequent consultation with JP and the other authors. The majority of the paper has been written by GK. All authors contributed to the article and approved the submitted version.

## Funding

We gratefully acknowledge the support of DTRAl -16-1-0002/Project # 1553695, eTASC - Empirical Evidence for a Theoretical Approach to Semantic Components; DARPA 15-18-CwC-FP-032 Communicating with Computers, C3 Cognitively Coherent Human-Computer Communication (sub from UIUC) and Elementary Composable Ideas (ECI) Repository (sub from SIFT); DARPA KAIROS FA8750-19-2-1004; and DARPA FA8750-18-2-0016-AIDA—RAMFIS: Representations of vectors and Abstract Meanings for Information Synthesis. Any opinions, findings, and conclusions or recommendations expressed in this material are those of the authors and do not necessarily reflect the views of DTRA, DARPA or the U.S. government.

## Conflict of Interest

The authors declare that the research was conducted in the absence of any commercial or financial relationships that could be construed as a potential conflict of interest.

## Publisher's Note

All claims expressed in this article are solely those of the authors and do not necessarily represent those of their affiliated organizations, or those of the publisher, the editors and the reviewers. Any product that may be evaluated in this article, or claim that may be made by its manufacturer, is not guaranteed or endorsed by the publisher.

## Footnotes

^1^Logical predicates do not have to have specific semantics. When we assign specific semantics to these predicates, they become “semantic” predicates. Therefore, semantic predicates are a subset of logical predicates. Throughout this paper, we use semantic predicates and logical predicates interchangeably, and they both refer to the same thing in the context of VerbNet.

^2^[+int_control] indicates a selectional preference scoping over this thematic role in this class. In other words, the agent in this class tends to have intentional control.

^3^The subclasses are marked as such in the XML files.

^4^Although such multi-verb expressions are not as common, they have been counted as VPC's in the literature (See the works cited in the text).
